# Deep learning-based artefact reduction in low-dose dental cone beam computed tomography with high-attenuation materials

**DOI:** 10.1098/rsta.2024.0045

**Published:** 2025-09-25

**Authors:** Hyoung Suk Park, Kiwan Jeon, J. K. Seo

**Affiliations:** ^1^ National Institute for Mathematical Sciences, Daejeon, Republic of Korea; ^2^ School of Mathematics and Computing (Computational Science and Engineering), Yonsei University, Seodaemun-gu, Seoul, Republic of Korea

**Keywords:** computed tomography, CBCT, metal artefact reduction, deep learning, implicit neural representation

## Abstract

This paper examines the current challenges in computed tomography (CT), with a critical exploration of existing methodologies from a mathematical perspective. Specifically, it aims to identify research directions to enhance image quality in low-dose, cost-effective cone beam CT (CBCT) systems, which have recently gained widespread use in general dental clinics. Dental CBCT offers a substantial cost advantage over standard medical CT, making it affordable for local dental practices; however, this affordability brings significant challenges related to image quality degradation, further complicated by the presence of metallic implants, which are particularly common in older patients. This paper investigates metal-induced artefacts stemming from mismatches in the forward model used in conventional reconstruction methods and explains an alternative approach that bypasses the traditional Radon transform model. Additionally, it examines both the potential and limitations of deep learning-based methods in tackling these challenges, offering insights into their effectiveness in improving image quality in low-dose dental CBCT.

This article is part of the theme issue ‘Frontiers of applied inverse problems in science and engineering’.

## 1. Introduction

This paper explores the key challenges in achieving high-quality imaging in low-cost, low-dose computed tomography (CT) systems, critically analysing the limitations of conventional methodologies from a mathematical perspective and suggesting future research directions. The growing focus on low-cost CT systems stems from the substantial economic burden associated with standard medical CT imaging [1], including high costs for machine purchase, operation and maintenance.

Over the past decade, the considerable reduction in the cost of dental cone beam CT (CBCT) systems has broadened access to advanced imaging technology for a wider range of dental practices. On the other hand, the increasing ageing population with metallic implants poses complex challenges, as traditional reconstruction techniques often result in severe metal-induced artefacts, leading to difficult issues in image distortion correction. From the perspective of image restoration, this underscores the critical need for innovative solutions to address these complex and ill-posed problems. This paper primarily focuses on dental CBCT to investigate the more complex ill-posed problems in CT imaging, although much of the discussion is equally relevant to standard CT systems.

In CT imaging, X-ray beams are directed through a patient positioned between an X-ray source and a detector. The gantry, which houses the X-ray source and detector assembly, rotates to emit beams from various directions. During this process, the detector captures projection data P that are a function of detector position and the beam-source angle. This projection data P are used to reconstruct a tomographic image, denoted by u, that represents the spatial distribution of the linear attenuation coefficient.

**Figure 1.**
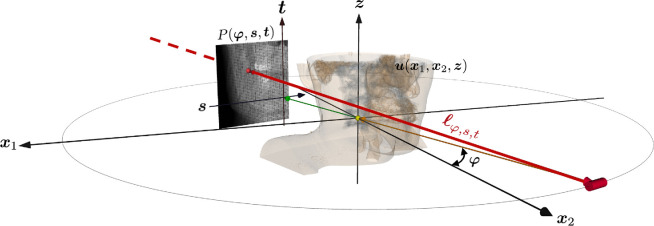
Low-dose dental CBCT and its truncated projection data. The figure illustrates a dental CBCT scan, where a cone-beam line $\ell_{\varphi ,s,t}$ passes through the patient's head, positioned between an X-ray source and a flat-panel detector housed in a rotating gantry. This rotation allows the X-ray beam to acquire projection data at various angles φ and detector positions (s,t). The projection data are truncated and offset due to the use of a small detector, designed to reduce costs and focus on a limited FOV.

To clearly and concisely explain the CT reconstruction mechanism from a mathematical perspective, we begin with a simplified two-dimensional model of fan-beam CT, corresponding to the midplane of the three-dimensional CBCT shown in figure 1. In the two-dimensional CT model, the detector captures projection data P(φ,s), where $\Theta_{\varphi }=\left(-\sin\;\varphi ,\cos\;\varphi \right)$ represents the beam-source angle, with φ ranging between 0 and 2π, and s indicates the detector position. The projection data P are defined as $P(\varphi ,s)=-\ln(\frac{I_{out}(\varphi ,s)}{I_{in}})$, where $I_{in}$ denotes the intensity of incoming X-ray photons, and $I_{out}(\varphi ,s)$, as measured by the detector, represents the intensity of X-ray photons that have traversed the patient’s body along the beam line $\ell_{\varphi ,s}$, which connects the beam source point at angle φ to the corresponding detector position s. The objective is to reconstruct a tomographic image u(x) at each pixel position $x=\left(x_{1},x_{2}\right)$ from the data P.

Traditionally, the reconstruction of a CT image relies on the assumption of a linear relationship $P(\varphi ,s)=\int_{\ell_{\varphi ,s}}u(x)dl_{x}$, representing the total attenuation experienced by the X-ray beam path $\ell_{\varphi ,s}$ as it traverses the body, where $dl_{x}$ is the line element. In this model, P corresponds to the fan-beam Radon transform of u, denoted as Ru. Under this ideal assumption, where P lies within the range of the Radon transform [2], standard reconstruction algorithms like fan-beam filtered back projection (FBP) are effective, as long as no high-density materials, such as metals, are present [3–6]. However, in reality, this linear model is inaccurate because the X-ray photons generated by X-ray tubes are polychromatic, and the attenuation coefficients of high-density materials vary significantly with photon energy. Hence, the data P obtained from an energy-integrated detector (EID), which is most commonly utilized in CT, are not linear with u at any fixed energy (see §2a). Due to this energy-dependent nature, a more accurate mathematical model for low-dose local CT can be formulated as


(1.1) $$\begin{array}{lcr} & P=SRu+\zeta_{u}+\zeta_{n}+\zeta_{m}, & \end{array}$$

where S represents a subsampling operator, $\zeta_{u}$ is a nonlinear artefact source dependent on the distribution of u (e.g. in the presence of metal, it encompasses beam hardening, photon starvation, scattering and other effects that result in sinogram inconsistency), $\zeta_{n}$ includes noise contributions such as electronic noise and quantum noise and $\zeta_{m}$ accounts for motion-induced sinogram errors, particularly in dental CBCT. These error factors cause P to deviate from the range of the operator SR, a situation referred to as *sinogram inconsistency*. Both the nonlinear sinogram inconsistency factor $\zeta_{u}$ and electron noise in $\zeta_{u}$ can be mitigated using a photon-counting detector (PCD). Its energy-resolving capability enables thresholding to reject low-energy noise while accurately counting X-ray photons based on their energy. However, this technology is not yet suitable for low-cost CT applications. Similarly, dual-energy CT (DECT) can improve beam-hardening correction by utilizing high- and low-energy projection datasets [7–9], but it also remains unsuitable for low-dose, low-cost CBCT systems. For detailed explanations, see §2b.

Since the terms $\zeta_{u}$, $\zeta_{n}$ and $\zeta_{m}$ in (1.1) are unknown, we typically deal with the linear underdetermined problem P=SRu, which is both inaccurate and ill-posed due to factors resembling a violation of the Nyquist criterion [10,11]. This often leads to either no solution or an infinite number of possible solutions, as the homogeneous equation SRu=0 has a high-dimensional null space. Notably, despite extensive research, addressing sinogram inconsistencies caused by high-density materials in CT scans has remained an unresolved challenge for over 50 years [12].

Regularized least-squares methods have been widely used to incorporate prior knowledge of CT image characteristics. Based on this framework, various iterative reconstruction algorithms have been developed, enabling adjustments at each step to reduce artefacts by incorporating image priors through the regularization [13–18]. However, the traditional regularized least-squares methods, which rely on handcrafted regularizations such as total variation and wavelet-sparsity constraints, often struggle to fully integrate prior knowledge that comprehensively captures both local and global interconnections among pixel elements. This limitation is particularly pronounced in low-dose dental CBCT scans involving dental implants, where data inconsistency, noise and undersampling synergistically lead to significant image degradation and the emergence of pronounced streaking artefacts. To effectively tackle these challenges, especially when nonlinear factors contribute to significant data inconsistencies, the strategic implementation of image priors is essential. See §2c for data characteristics and detailed analysis.

Many ill-posed problems can be transformed into well-posed ones by restricting the set of permissible solutions to a compactly defined subset, denoted as M. In this context, M represents an unknown manifold for dimensionality reduction. Unfortunately, accurately defining the constrained set M presents a significant challenge due to the complex and high-dimensional nature of medical images, where critical features and variations are intricately intertwined. While identifying M in high-dimensional medical imaging data is highly challenging with current artificial intelligence methodologies, empirical evidence suggests that supervised deep learning (DL) architectures, when trained with appropriate paired data, have demonstrated remarkable capabilities in deriving an image restoration function $f:u^{♯}\mapsto u\in M$. Here, $u^{♯}$ is regarded as a reconstructed CT image obtained using conventional methods, such as the regularized least-squares method. This approach is particularly effective when $u^{♯}$ reflects the diagnostic features and anatomical structures of the true medical image u∈M. However, despite the efficiency of supervised learning, obtaining paired data in clinical settings for dental CBCT remains extremely difficult. Deep generative models offer a notable advantage when working with unpaired datasets; however, they have limitations in capturing the subtle variations required for detecting rare pathologies. See §4a for a detailed explanation of the limitations of deep generative models.

Despite significant advances in DL, training a neural network to effectively map directly from P to u∈M remains a considerable challenge in practice. In particular, learning the Radon and Fourier transforms in high-dimensional medical imaging is extremely difficult, even though some research on lower-dimensional images has shown promising results. Additionally, learning transformations within the sinogram space, such as sinogram correction and inpainting to map from P to $P_{*}=Ru$ for some u∈M, are extremely challenging due to the difficulties in maintaining sinogram consistency, which is crucial as it represents the range space of the Radon transform.

Even when P is fully sampled data and noise-free, a discrepancy still exists between P and $RR^{-1}P$ due to the polychromatic nature of X-ray beams. Here, $R^{-1}$ denotes the FBP algorithm, which is utilized to perform the inverse Radon transform. According to the Hilbert projection theorem, P is decomposed into $P_{\text{range}}$ in the range space of R and its orthogonal complement $P_{\text{range}}^{⊥}$. Therefore, $R^{-1}P=R^{-1}P_{\text{range}}$, which exhibits severe streaking artefacts in the presence of high-attenuation materials. For a thorough analysis, see §2c. Consequently, in scenarios involving high-attenuation materials, it would be prudent to consider alternative methods to $R^{-1}$.

Recently, an alternative method based on implicit neural representations (INRs) has been proposed to circumvent the reliance on traditional backprojection $R^{-1}$ [19–21]. In this approach, a neural network is trained to implicitly represent CT images by incorporating the Beer–Lambert law and beam-hardening effects into its loss function. The network takes spatial coordinates (e.g. pixel positions) as inputs and produces corresponding energy-independent quantities, such as the attenuation coefficient at a fixed energy level or density. Experimental results suggest that INRs have the potential to effectively address nonlinear factors associated with model mismatch, circumventing the need for backprojection. However, despite its considerable potential, further improvements in robustness and the ability to handle severely corrupted data, such as that caused by photon starvation and patient movement during scanning, are essential for clinical application. See §4b,c for detailed explanations.

This paper provides a comprehensive analysis of the potential and limitations of existing methods, with a focus on key challenges and practical and clinical applications. Acknowledging the need for extensive discussion in clinical settings, we aim to concisely cover the essential aspects to guide future research, taking into account the rapid increase in the ageing population with metal implants and the economic issues posed by costly advanced technologies contributing to the healthcare bubble.

## 2. Mathematical framework for low-dose and low-cost computed tomography

### (a) Forward computed tomography model with energy-integrated detector

The mathematical two-dimensional forward model for CT imaging is to provide the relationship between the two-dimensional cross-sectional CT image u(x) (the linear attenuation coefficient at pixel position $x=\left(x_{1},x_{2}\right)$) and the X-ray projection data P(φ,s). The projection data P, measured from an EID, can be expressed in integral form as follows:


(2.1) $$\begin{array}{lcr} & P(\varphi ,s)=\int \eta (E)P_{E}(\varphi ,s)\;dE, & \end{array}$$

where $P_{E}$ denotes the X-ray data at energy level E, and η(E) represents the fractional energy distribution of photons at energy E. The support of η(E) is defined within the interval [0,200] KeV, and ∫η(E)dE=1. The attenuation coefficient u for different materials (such as tissues, bones and metals) varies to different extents with energy E. For denser materials like metal, the change in u with E below 70 keV is significant, whereas for softer tissues, the variation is small. To emphasize the dependency on E, we denote the attenuation coefficient as $u_{E}$.

The fundamental principle guiding CT imaging, the Beer–Lambert law, is expressed as follows:


(2.2) $$P(\varphi ,s)=-\ln\left(\int \eta (E)e^{-\int_{\ell_{\varphi ,s}}u_{E}(x)dl_{x}}dE\right).$$

The goal of CT imaging is to reconstruct u from P, aiming for u to approximate $u_{E_{0}}$ at a fixed energy $E_{0}$, as it is not feasible to reconstruct $u_{E}$ across multiple energy levels. In this reconstruction, u must ensure that the projection data P(φ,s) closely match the line integral of u along the ray path $\ell_{\varphi ,s}$, as described by the Radon transform


(2.3) $$\begin{array}{lcr} & P(\varphi ,s)\approx \int_{\ell_{\varphi ,s}}u(x)\;dl_{x}=Ru(\varphi ,s), & \end{array}$$

where $dl_{x}$ is the length element. However, this standard CT model exhibits a mismatch with the true model in (2.1), primarily due to the polychromatic nature of the X-ray beam and the energy-dependent variations in attenuation. Additionally, the projection data are influenced by various noise factors, including quantum noise, electronic noise and scatter radiation noise. Before delving into reconstruction methods, it is essential to first understand these noise factors.

The mathematical three-dimensional forward model for low-dose dental CBCT closely mirrors the two-dimensional model described earlier, though the reconstruction method differs and introduces additional challenges. As shown in figure 1, this model defines the relationship between the three-dimensional image u(x,z) at the point $(x,z)=(x_{1},x_{2},z)\in {\mathbb{R}}^{3}$ and the CBCT projection data P(φ,s,t), where (s,t) represents the scaled planar detector position. Similar to (2.2), the relationship between P and u is expressed as


(2.4) $$P(\varphi ,s,t)=-\ln\left(\int \eta (E)e^{-\int_{\ell_{\varphi ,s,t}}u_{E}(x,z)dl_{x}}dE\right),$$

where $\ell_{\varphi ,s,t}$ is the cone-beam line associated with (φ,s,t). The standard CBCT reconstruction algorithm is the FDK method, developed by Feldkamp *et al*. [22]. This FDK method can be regarded as an empirical three-dimensional extension of the standard two-dimensional FBP algorithm. Most dental CBCT scans can be considered as local CT due to the truncated field of view (FOV), as shown in figure 1. This FOV truncation is caused by the use of small detectors, driven by the significant manufacturing costs associated with the X-ray detector component of a CBCT device. The challenges related to dental CBCT will be discussed in §4.

### (b) Mitigating model mismatch in computed tomography: limitations of photon counting and dual-energy computed tomography

This subsection concentrates solely on the model mismatch resulting from the nonlinear term $\zeta_{u}$, while neglecting the contributions of $\zeta_{n}$ and $\zeta_{m}$ in (1.1). The mismatch between the standard model in (2.3) and the true model in (2.1) can be expressed as


(2.5) $$\zeta_{u}(\varphi ,s,t)=-\ln\left(\int \eta (E)e^{-\int_{\ell_{\varphi ,s,t}}(u_{E}(x)-u(x))dl_{x}}dE\right).$$

This mismatch can be mitigated by using photon counting CT (PCCT) scanners, which utilize energy-resolving detectors capable of distinguishing between photons of different energies. Unlike conventional X-ray detectors, which first convert X-ray photons to fluorescent light and then to electrical signals, PCDs directly convert X-ray photons into electrical signals. The main challenges for PCCT include manufacturing low-cost detectors with a low density of imperfections, achieving high performance at elevated count rates and maintaining good spectral fidelity [23]. Despite several recent advancements, PCCT scanners remain significantly more expensive than conventional CT scanners, primarily due to the complex manufacturing processes of the detectors and the high operational and maintenance costs [24,25]. Consequently, the widespread replacement of conventional CT systems with PCCT technology in clinical settings is unlikely in the near future. Similarly, DECT can help address the mismatch in (2.5) by using dual projection data $P_{1}$ and $P_{2}$ from two different energy distributions to acquire the combined projection data $P_{\text{DE}}=g(P_{1},P_{2})$, where g is a function designed to reduce artefacts by integrating information from both energy levels. Since today’s CT detectors integrate all the fluorescent light intensities produced by photon interactions in the scintillator during a readout interval without capturing individual photon energies, current DECT approaches either use entirely dual X-ray sources and dual-layer detectors or acquire projection data at different time points. Due to these complexities, DECT is not suitable for low-dose and low-cost CT applications, as it leads to higher radiation exposure and increased costs [9]. Therefore, this paper does not consider PCCT and DECT technologies.

### (c) Challenges in low-dose dental cone beam computed tomography: noise, artefacts and image degradation

Projection data in standard CT and dental CBCT differ significantly due to differences in scanning geometry, FOV and detector placement. Dental CBCT systems typically use small flat-panel detectors with slower scanning speeds (ranging from 7 to 24 s), offering a much lower cost and a compact design that requires significantly less space compared with standard CT [26,27]. However, this design results in projection data with a truncated FOV and asymmetry due to the offset detector configuration, which is more commonly used in clinical practice than centred CBCT, as it enables an extended effective FOV without increasing detector size, making it suitable for full-arch and maxillofacial imaging. Additionally, dental CBCT systems experience increased Compton scattering due to the detector’s close proximity to the patient’s face and cone-beam artefacts caused by violations of Tuy’s condition [28]. These differences in dental CBCT not only lead to additional inconsistent sinogram data compared with standard CT but also cause inaccuracies in Hounsfield units (HU) [29].

Specifically, let $T_{\text{ fw}}$ represent a linear X-ray transform, such as a truncated and offset version of the fan-beam Radon transform or the cone-beam transform, that maps a CT image u to its corresponding sinogram P


(2.6) $$\begin{array}{lcr} & T_{\text{ fw}}:u\in {\mathbb{R}}^{n_{\text{image}}}\mapsto P\in H, & \end{array}$$

where $n_{\text{image}}$ denotes the number of pixels (or voxels) in the image space, and $H:={\mathbb{R}}^{n_{\text{view}}\times n_{\text{de}}}$ is the sinogram space, with $n_{\text{view}}$ representing the number of views and $n_{\text{de}}$ the number of detector cells.

#### (i) Impact of sinogram inconsistency on computed tomography image reconstruction

In dental CBCT, the sinogram P is mostly inconsistent, meaning that $P\notin H_{\text{ range}}:=\left\{T_{\text{ fw}}u:u\in {\mathbb{R}}^{n_{\text{image}}}\right\}$, i.e. P does not reside within the range space of the forward operator $T_{\text{ fw}}$. This inconsistency is especially exacerbated by the presence of high-attenuation materials like metals, leading to pronounced metal-induced streaking artefacts. To be more precise, if we treat the sinogram space H in (2.6) as a Hilbert space with the standard Euclidean norm, it can be decomposed as


(2.7) $$H=H_{\text{ range}}⊕H_{\text{ range}}^{⊥},$$

where $H_{\text{ range}}^{⟂}$ denotes the orthogonal complement of $H_{\text{ range}}$, and ⊕︎ represents the orthogonal direct sum. Consequently, P can be expressed as


(2.8) $$P=P_{\text{ range}}+P_{\text{ range}}^{⊥},$$

where $P_{\text{ range}}\in H_{\text{ range}}$ and $P_{\text{ range}}^{⟂}\in H_{\text{ range}}^{⟂}$.

**Figure 2.**
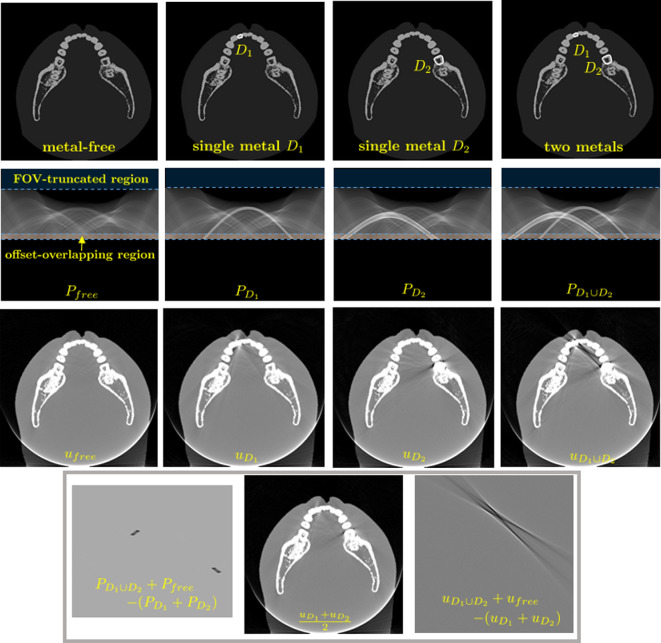
Phantom experiment illustrating the combined nonlinear effects associated with two metals $D_{1}$ and $D_{2}$. The first row shows four phantom images: metal-free, containing single metal $D_{1}$, single metal $D_{2}$ and two metals $D_{1}\cup D_{2}$. The second row presents the corresponding sinograms. The third row displays the reconstructed images $u_{\text{ free}},u_{D_{1}},u_{D_{2}},u_{D_{1}\cup D_{2}}$ using the FBP method. The fourth row highlights the nonlinear beam-hardening effects, showing the differences $P_{D_{1}\cup D_{2}}+P_{\text{ free}}-(P_{D_{1}}+P_{D_{2}})$ and $u_{D_{1}\cup D_{2}}+u_{\text{ free}}-(u_{D_{1}}+u_{D_{2}})$. From the image of $u_{D_{1}\cup D_{2}}$, we observe combined streaking artefacts resulting from metal–bone and metal–metal interactions, as well as FOV truncation.

Systematic artefacts, particularly those resulting from beam-hardening effects, are related to the difference $P-P_{\text{ range}}$. For ease of explanation, consider the classical case of two-dimensional CT. The orthogonal complement $P_{\text{ range}}^{⟂}$ can be computed as $P_{\text{ range}}^{⟂}=P-RR^{-1}P$ and satisfy $R^{-1}P_{\text{ range}}^{⟂}=0$. Hence, the FBP reconstruction $R^{-1}P$ is the same as $R^{-1}P_{\text{ range}}$, where P is replaced by the corresponding $P_{\text{ range}}$. Typically, metal-induced beam hardening in P is confined to a localized region, whereas this local beam-hardening effect becomes globally spread in $P_{\text{ range}}$ due to the orthogonal projection onto the range space. Consequently, in the process of CT reconstruction, orthogonally projecting P onto the range space causes data distortion to spread across the entire domain, leading to the formation of structural artefacts. This effect is demonstrated in figure 2.

In the context of data-fidelity minimization, the optimization essentially deals with $P_{\text{ range}}$ rather than P because


(2.9) $$\underset{u}{\mathrm{argmin}}{\left\|P-T_{fw}u\right\|}_{\ell_{2}}=\underset{u}{\mathrm{argmin}}{\left\|P_{\text{range }}-T_{fw}u\right\|}_{\ell_{2}}.$$

It should be noted that $T_{\text{ fw}}$ maps even a single-pixel image to a global sinusoidal curve in the sinogram space H. From this perspective, conversely, if there is an inconsistency in a single pixel in P, a global change occurs in $P_{\text{ range}}$ during the projection of P onto the range space of $H_{\text{ range}}$. The next §2c(ii) will provide an intuitive example using a toy model.

Ideally, it would be best to correct the local inconsistencies in P during a preprocessing step. However, directly correcting the sinogram without backprojection combined with some form of regularization in the image space is extremely challenging, as maintaining sinogram consistency is difficult. Interestingly, even if a reconstructed CBCT image is severely corrupted by metal artefacts, a panoramic image reprojected from the corrupted CT image appears free of metal artefacts due to the integration process, which cancels out dark and bright streaking artefacts [30]. This gives hope that systematic artefacts can be managed when prior information is properly incorporated and constrained.

Let us briefly discuss the issue of uniqueness in the ill-posed problem $T_{\text{ fw}}u=P$ under the assumption that $P=P_{\text{ range}}$ (i.e. high-quality undersampled data without beam-hardening effects), as it has infinitely many mathematical solutions. Imagine that M represents a low-dimensional manifold defined by the set of physically meaningful and naturally occurring CT images. The operator $T_{\text{ fw}}$ is said to satisfy the M-restricted isometry property (RIP) condition with respect to the constrained set M if there exists a positive constant C such that


(2.10) $$\frac{1}{C}\left\|u-u^{\prime }\right\|\leq \left\|T_{fw}u-T_{fw}u^{\prime }\right\|\leq C\left\|u-u^{\prime }\right\|\;\text{ for all }u,u^{\prime }\in M.$$

This RIP condition guarantees that the problem $T_{\text{ fw}}u=P$ has a unique solution when constrained to u∈M. Specifically, if $u,u^{\prime }\in M$ satisfy $T_{\text{ fw}}u=P=T_{\text{ fw}}u^{\prime }$, then $\|T_{\text{ fw}}u-T_{\text{ fw}}u^{\prime }\|=0$, implying $\|u-u^{\prime }\|=0$ due to the lower bound $\frac{1}{C}\|u-u^{\prime }\|\leq \|T_{\text{ fw}}u-T_{\text{ fw}}u^{\prime }\|$. This concept of M-RIP is a variation of RIP, originally introduced and utilized in prior works [31,32]. This M-RIP condition allows us to select a practical solution from the infinitely many mathematical solutions. With recent advancements in DL techniques, the ill-posed problem $T_{\text{ fw}}u=P$ can be effectively handled using suitable training data that somewhat act as a data-driven prior or M. Although this approach has no rigorous evidence and M is unknown, this ill-posedness can be managed using DL techniques.

#### (ii) Illustrative example of local sinogram inconsistency leading to global artefacts

Consider a toy model with bichromatic energies of 64 and 80 keV, where the fractional energy is given by $\eta (E)=\frac{1}{2}\delta (E-64)+\frac{1}{2}\delta (E-80)$. For simplicity, we adopt a parallel-beam geometry. Suppose the image to be reconstructed is represented as a 3×3 pixel matrix


(2.11) $$\begin{array}{lcr} & \left(\begin{array}{ccc} u_{1,1} & u_{1,2} & u_{1,3}\\ u_{2,1} & u_{2,2} & u_{2,3}\\ u_{3,1} & u_{3,2} & u_{3,3} \end{array}\right). & \end{array}$$

In this scenario, let us assume that $u_{2,1}$ and $u_{2,3}$ correspond to metal, while the remaining pixels represent air. Consequently, we expect the reconstructed image to take the form


(2.12) $$\begin{array}{lcr} & \left(\begin{array}{ccc} 0 & 0 & 0\\ c & 0 & c\\ 0 & 0 & 0 \end{array}\right), & \end{array}$$

where c is a constant associated with the attenuation coefficient of the metal. It is measured at three different detector positions (s=1,2,3) for three views ($\varphi =0,\frac{\pi }{4},\frac{\pi }{2}$). Given that the attenuation coefficients for the metal are 64 at *E* = 64 keV and 5 at *E* = 80 keV, the conventional CT reconstruction problem can be formulated by solving the following system of equations:


(2.13) $$\left\{ \begin{array}{llll} u_{1,1}+u_{2,1}+u_{3,1} & = & P(0,1) & =5.7\\ u_{1,2}+u_{2,2}+u_{3,2} & = & P(0,2) & =0\\ u_{1,3}+u_{2,3}+u_{3,3} & = & P(0,3) & =5.7\\ u_{2,1}+u_{3,2} & = & P(\pi /4,1) & =5.7\\ u_{1,1}+u_{2,2}+u_{3,3} & = & P(\pi /4,2) & =0\\ u_{1,2}+u_{2,3} & = & P(\pi /4,3) & =5.7\\ u_{3,1}+u_{3,2}+u_{3,3} & = & P(\pi /2,1) & =0\\ u_{2,1}+u_{2,2}+u_{2,3} & = & P(\pi /2,2) & =10.7\\ u_{1,1}+u_{1,2}+u_{1,3} & = & P(\pi /2,3) & =0 \end{array}\right..$$

Here, the value 10.7 is derived from 10.7≈−log⁡(0.5exp⁡(−64×2)+0.5exp⁡(−5×2)), and 5.7 is calculated as 5.7≈−log⁡(0.5exp⁡(−64×1)+0.5exp⁡(−5×1)). The standard reconstruction algorithm is to find $u^{†}$ such that


(2.14) $$u^{†}=\mathrm{argmin}\|Au-P{\|}_{\ell_{2}}^{2},$$

where A is the 9×9 matrix matrix representing the Radon transform in (2.13) and u is the vectorized form of the 3×3 pixel matrix in (2.11). The reconstructed image, computed using the formula $u^{†}=(A^{T}A{)}^{-1}A^{T}P$, is given by


(2.15) $$\begin{array}{lcr} & \left(\begin{array}{ccc} -1.0 & 2.2 & 0.4\\ 6.8 & 2.5 & 6.3\\ 0.2 & -0.5 & 0.7 \end{array}\right)\neq \left(\begin{array}{ccc} 0 & 0 & 0\\ c & 0 & c\\ 0 & 0 & 0 \end{array}\right). & \end{array}$$

It is important to note that the reconstructed image $u^{†}$ significantly deviates from the expected solution in (2.12) due to the backprojection process $A^{T}P$. This discrepancy arises from a single mismatch observed in the eighth equation of (2.13), where P(π/2,2)=10.7≠2×5.7.

#### (iii) Nonlinear impact of metal-induced artefacts in dental cone beam computed tomography

We now illustrate image degradation in CBCT caused by multiple factors, including metal-induced inconsistencies, data truncation (commonly encountered in dental CBCT) and noise. These effects are demonstrated through realistic simulations using the standard FDK algorithm.

To simplify the explanation, we focus on the midplane of a dental CBCT model, restricting P(φ,s,t) to the detector position t=0. In this context, the forward projection operator $T_{\text{ fw}}$ can be represented as a composition of the Radon transform $R=T_{\text{ fw}}$ and a data-rebinning operator that converts fan-beam projection data into a parallel-beam sinogram [33].

For the simulation, as shown in figure 2, we use realistic CBCT data $P_{\text{ free}}$ from a patient without metallic implants. By applying the FBP algorithm to $P_{\text{ free}}$, we obtain a metal artefact-free image, denoted by $u_{\text{ free}}$. Next, we insert two metal objects at different positions within $u_{\text{ free}}$, occupying regions $D_{1}\cup D_{2}$, and generate the projection data $P_{D_{1}\cup D_{2}}$ using the physical model described in (2.2). Reconstructing the image from $P_{D_{1}\cup D_{2}}$ using the standard FBP algorithm, we obtain $u_{D_{1}\cup D_{2}}$, which exhibits significant metal-induced artefacts.

To explore the nonlinear effects associated with the presence of two metals, we also generate the projection data $P_{D_{1}}$ and $P_{D_{2}}$, corresponding solely to regions $D_{1}$ and $D_{2}$, respectively. The reconstructed images from $P_{D_{1}}$ and $P_{D_{2}}$ are denoted as $u_{D_{1}}$ and $u_{D_{2}}$. Interestingly, the combined image $u_{D_{1}}+u_{D_{2}}$ shows relatively fewer metal artefacts compared with $u_{D_{1}\cup D_{2}}+u_{\text{ free}}$.

This difference can be understood by considering the X-ray beams passing through both metal objects. Along these paths, $P_{D_{1}\cup D_{2}}+P_{\text{ free}}$ experiences more severe beam hardening compared with $P_{D_{1}}+P_{D_{2}}$. As a result, the sinogram mismatches in $P_{D_{1}\cup D_{2}}$ are more pronounced along rays intersecting both $D_{1}$ and $D_{2}$. During the backprojection process, these mismatches spread across the entire sinogram, disrupting its global structure and generating streaking and shadowing artefacts. This adverse effect is inherent to the minimization process described in (2.14).

## 3. Limitations of conventional reconstruction methods in dental cone beam computed tomography

Most metal artefact reduction (MAR) techniques developed over the last four decades have been designed for standard CT, not specifically for the unique challenges of dental CBCT [16,34–45]. As mentioned in the previous section, we exclude discussions on data acquisition improvement methods such as higher X-ray energy, DECT and photon-counting CT, as they are not suitable for low-cost, low-dose dental CBCT.

Many commercial MAR approaches are based on sinogram correction techniques, which correct or replace unreliable projection data, particularly within metal traces, before reconstruction. These include SEMAR (Toshiba Medical Systems) [46], O-MAR (Philips Healthcare) [47], iMAR (Siemens Healthcare) [48] and Smart MAR (GE Healthcare) [49], which implement these techniques either directly or iteratively. These methods typically segment the metal region using thresholding operations and then apply either interpolation-based inpainting (faster) or prior image-based sinogram completion (more computationally demanding) to replace metal-affected regions. A prior image is often generated by segmenting the initial CT image into different tissue classes, such as air, soft tissue and bone, using multi-thresholding techniques. These types of prior images are used for efficient interpolation in a tissue-normalized sinogram, mitigating metal-induced artefacts [48,50,51]. This technique was initially introduced in normalized MAR [42]. However, the effectiveness of these approaches largely depends on the accuracy of segmentations and the quality of the prior image [52,53]. In particular, the threshold-based segmentation techniques struggle with inaccuracies in HU values in dental CBCT, as discussed in §2c. Furthermore, iterative sinogram correction, as used in iMAR, refines sinogram data through an iterative process of reprojection and backprojection but faces challenges in dental CBCT due to high computational costs and incomplete projection data.

These MAR methods employ a hybrid approach, integrating sinogram correction with image post-processing. Sinogram correction addresses the sources of major metal-induced artefacts at the projection level, while image post-processing enhances the final reconstructed image by removing residual artefacts and improving clarity. Typically, the corrected sinogram is modified only in the metal-affected regions, while the exterior sinogram (the portion outside the metal trace) generally remains unchanged. Note that sinogram data inconsistencies arise not only from metals but also from teeth and bones. Thus, in the image post-processing stage, which aims to further refine MAR-corrected images, various image denoising methods can be combined with sinogram denoising to address residual artefacts resulting from imperfections in sinogram correction, scattering and other factors.

In the remainder of this section, we focus on image post-processing. For simplicity of notation, we redefine P as the corrected sinogram, which remains unchanged outside the metal-affected regions. Conventional methods typically rely on a regularized least-squares approach, expressed as


(3.1) $$u_{*}=\underset{u}{\mathrm{argmin}}{\left\|P-T_{fw}u\right\|}_{\ell_{2}}^{2}+\lambda \mathrm{Reg}(u),$$

where Reg(u) is a regularization term and λ is the regularization parameter. We note that the target u to be reconstructed is typically expected to satisfy a weak fidelity condition, meaning that $\|P-T_{\text{ fw}}u{\|}_{\ell_{2}}^{2}$ is not very small due to noise and residual model mismatch in P. As a result, selecting a suitable regularization term Reg(u) as a form of image prior information is essential, as it plays a critical role in suppressing noise and artefacts.

**Figure 3.**
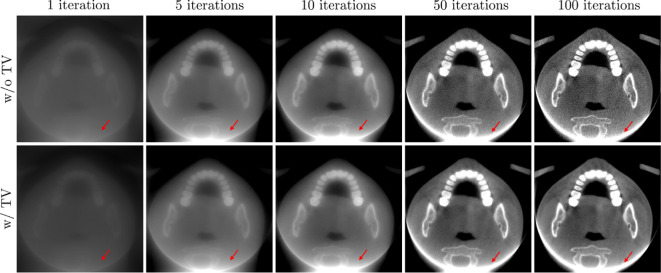
Results of the iterative reconstruction model in (3.1) over iterations for three-dimensional dental CBCT with a truncated FOV. The first and second rows present the results with and without total variation regularization, respectively. As iterations progress, the discrepancy between the actual projection and the forward projection within the truncated FOV accumulates regardless of regularization, leading to significant degradation in the reconstructed CT image quality, as indicated by the red arrows.

Various denoising methods have been developed using sparsity-based cost functions as image priors, enforcing sparsity in different domains. Total variation denoising exploits sparsity in the gradient domain [54–56]; wavelet-based denoising [57,58] leverages sparsity in the wavelet domain; dictionary learning-based denoising [59] relies on sparse representation in a learned basis; Iterative Shrinkage-Thresholding Algorithm (ISTA) [60,61] and FISTA (fast ISTA) [54,62] are widely used iterative approaches that employ soft-thresholding operators to solve the Lasso problem, formulated as a sparsity-based ($\ell^{1}$-regularized) optimization; and plug-and-play ISTA (PnP-ISTA) [63] extends ISTA by replacing the shrinkage step with a learned or predefined denoiser. These sparsity-inspired regularization methods effectively reduce noise, preserve edges and perform well in the absence of metal, even in low-dose CT. However, their application to dental CBCT poses significant challenges due to complex artefact patterns, as described in (1.1), along with FOV truncation and missing projection data. Notably, the adjoint of the forward map $T_{\text{ fw}}$ appears in the gradient of $\|P-T_{\text{ fw}}u{\|}_{\ell_{2}}^{2}$, and its application during the reconstruction process introduces structured noise that is difficult to mitigate using the regularization term Reg(u), as discussed in §2c. Figure 3 illustrates the image degradation effects caused by a truncated FOV.

## 4. Deep learning-based methods and challenges in three-dimensional maxillofacial cone beam computed tomography imaging

This section examines DL models for CT image denoising. DL has recently emerged as a transformative approach, offering a compelling alternative to traditional denoising methods. Regardless of how advanced a DL model is, it cannot resolve cases where two different clean CBCT images produce the same data, meaning that if RIP condition (2.10) is violated, the problem remains fundamentally unsolvable. In other words, DL models cannot solve overly underdetermined problems.

Typically, DL-based reconstruction algorithms start with a preliminary CT image $u_{‡}$ reconstructed from sinogram data P using a conventional algorithm like FDK, due to the challenges of correcting the sinogram directly in the dental CBCT environment. Here, $u_{‡}$ can be expressed as:


(4.1) $$u_{‡}(x,z)=\int_{0}^{2\pi }\int K(x,z;\varphi ,s)P(\varphi ,s,t_{\varphi ,x,z})dsd\varphi ,$$

where K(x,z;φ,s) is a reconstruction kernel and $t_{\varphi ,x,z}$ represents the vertical detector position corresponding to φ,x,z. See [11,22] for the explicit formula of the FDK algorithm. Conceptually, DL algorithms aim to learn a θ-parameterized neural network $f_{\theta }:u_{‡}\mapsto \hat{u}$, where the input is a degraded image $u_{‡}$, and the output is the corresponding restored image $\hat{u}$, with the objective that the predicted image $\hat{u}=f_{\theta }(u_{‡})$ closely approximates a ground truth image $u_{*}$. In CBCT image reconstruction, obtaining detailed 3D representations of a patient’s teeth, jawbones and overall dental anatomy is essential for advanced dental care [11]. Preserving small anomalous features while mitigating metal artefacts is critical for clinical accuracy.

DL models for dental CBCT can be trained using either supervised or unsupervised methods. While supervised learning is highly efficient, obtaining paired data (i.e. input-out pairs for $f_{\theta }$) in the clinical dental CBCT environment is nearly impossible. Based on our experience, generating semi-synthetic data by combining real CBCT images with numerically simulated metal artefacts of varying shapes and positions is an extremely laborious and cumbersome process, especially in a three-dimensional setting [11]. Although generative networks can make simulated data appear similar to real CBCT data, subtle differences remain, making them unreliable for paired training in clinical settings. Therefore, supervised learning using paired data remains challenging for further improvement.

### (a) Deep learning-based image enhancement methods

Sparsity-inspired denoising techniques, as discussed in §3, have been combined with DL models to leverage advantages such as data-driven optimization instead of fixed bases or dictionaries, adaptive thresholding instead of handcrafted shrinkage and unrolled iterative optimization, among others. These hybrid approaches include PnP-DL [63], ISTA-Net [64], learned proximal networks [65], learned primal-dual reconstruction [66] and Deep Dictionary Learning [67]. However, these hybrid methods face practical challenges in dental CBCT applications. Some methods are limited by the lack of paired training data, and most present significant clinical implementation challenges due to high memory and computational requirements. Although data-driven denoisers in PnP-DL can be trained on unpaired data and effectively address Gaussian noise, they often struggle with more complex, systematic artefacts associated with scattering and beam-hardening, especially in cases involving multiple metallic objects, as discussed previously.

DL algorithms for MAR can be categorized into three approaches: image-domain learning [68–72], projection-domain learning [73–75] and dual-domain learning [76,77]. Image-domain learning starts with a preliminary CT image $u_{‡}$, reconstructed from a sinogram data P using a basic conventional algorithm like FDK, and aims to learn a θ-parameterized neural network $f_{\theta }:u_{‡}\mapsto \hat{u}$, as previously mentioned. This approach aims to mitigate metal artefacts in reconstructed images by leveraging DL’s data-driven capability to capture anatomical details while reducing metal artefacts. However, their effectiveness heavily depends on the quality of the input image, which is often degraded by metal-induced artefacts such as streaking and shadowing. Consequently, they face challenges in effectively restoring severely corrupted CBCT images containing multiple metal objects. Several studies have investigated using the restored image $\hat{u}$ as an image prior to replacing metal-corrupted sinograms [68,72]. These methods require accurate metal segmentation; however, as previously discussed, distinguishing metal from severe artefacts in CBCT images remains highly challenging. The projection-domain learning approach directly corrects sinogram inconsistencies using a neural network $g_{\theta }:P\mapsto \hat{P}$, where $\hat{P}$ represents the restored sinogram. Unlike image-domain approaches, this approach does not rely on metal segmentation. However, its application has been restricted to patient-specific implant models, and extending it to CBCT scans with implants of various shapes and materials remains challenging [74]. In dual-domain learning, two networks, $f_{\theta }$ and $g_{\theta }$, operate separately in the sinogram and image domains but are trained in an end-to-end manner. The sinogram enhancement network is designed to correct metal-corrupted sinograms by employing an inpainting loss on the metal trace and a sinogram consistency loss [76]. Since the final reconstructed image is generated by the image enhancement network, data fidelity may be compromised, potentially leading to anatomical distortions. To mitigate this issue, Yu *et al.* [77] proposed a dual-domain learning framework that first generates a high-quality prior image with reduced metal artefacts. The forward projection of this prior image is then utilized for sinogram completion, specifically along the metal trace. This approach ensures that only the metal-affected regions in the sinogram are modified, thereby preserving overall data fidelity in the final reconstructed image. However, optimizing a three-dimensional MAR model that simultaneously learns both sinogram correction and image refinement is computationally demanding and poses a significant memory burden.

Since supervised learning is challenging in clinical dental CBCT due to the difficulty of collecting paired datasets of metal-free and metal-corrupted CBCT scans, we explore generative models as an alternative unsupervised learning approach. Let $p_{*}$ denote the distribution of ground truth images and $p_{\theta }$ represent the distribution of the reconstructed images (i.e. $\hat{u}$) using the neural network $f_{\theta }$ depending on the parameter θ. We denote unpaired datasets by ${\text{Data}}_{‡}=\left\{u_{‡}^{\left(1\right)},\cdots \;,u_{‡}^{\left(N_{‡}\right)}\right\}$ (a sample distribution of degraded images) and ${\text{Data}}_{*}=\left\{u_{*}^{\left(1\right)},\cdots \;,u_{*}^{\left(N_{*}\right)}\right\}$ (a sample distribution of reference images). The use of deep generative models, such as generative adversarial networks (GANs) or diffusion models (DMs), is particularly advantageous when working with unpaired datasets. These models leverage a parametric prior $p_{\theta }$, enabling them to generate highly realistic data. To learn the restoration network $f_{\theta }$, the optimal parameters θ can be determined through a loss minimization process such as


(4.2) $$\hat{\theta }=\underset{\theta }{\mathrm{argmin}}E_{u_{‡}∼p_{‡}}\left(-\log p\left(u_{‡}|f_{\theta }\left(u_{‡}\right)\right)+\lambda \mathrm{div}\left(p_{*},p_{\theta }\right)\right),$$

where $p(u_{‡}|f_{\theta }(u_{‡}))$ is a likelihood serving as the data fidelity, λ>0 acts similarly to the regularization parameter in (3.1) and $\text{div}(p_{*},p_{\theta })$ represents a divergence (e.g. Kullback–Leibler divergence or Pearson $\chi^{2}$ divergence) used to guide the learning of $p_{\theta }$ from ${\text{Data}}_{*}$, in order to approximate $p_{u_{*}}$.

Although deep generative models have demonstrated remarkable performance compared with conventional methods, their major drawback for use in medical practice is the difficulty in balancing a trade-off between perceptual quality (associated with the divergence between $p_{\theta }$ and $p_{*}$) and reconstruction accuracy (associated with diagnostic semantic similarity between $f_{\theta }(u_{‡})$ and $u_{*}$) [78].

In the context of GANs, the loss minimization from (4.2) can be formulated as an adversarial framework incorporating a supplementary discriminator network D


(4.3) $$\begin{array}{lcr} & \underset{\theta }{\text{argmax}}\underset{D}{\text{argmin}}E_{u_{*}∼p_{*}}[-\log\;D(u_{*})]+E_{u_{‡}∼p_{‡}}[-\log(1-D(f_{\theta }(u_{‡})))], & \end{array}$$

and the log likelihood $-\log\;p(u_{‡}|f_{\theta }(u_{‡}))$ often uses a weak fidelity $\|f_{\theta }(u_{‡})-u_{‡}{\|}^{2}$ (this weak fidelity, unfortunately, may be the best option available due to the absence of paired data) [79–83]. To better understand the model’s dependence on training data and concerns around memorization, we can express the discriminator loss as $-\frac{1}{N_{*}}\sum_{n=1}^{N_{*}}\log D(u_{*}^{\left(n\right)})-\frac{1}{N_{‡}}\sum_{m=1}^{N_{‡}}\log\left(1-D(\;f_{\theta }(u_{‡}^{\left(m\right)}))\right)$, and the generator loss is given by $-\frac{1}{N_{‡}}\sum_{m=1}^{N_{‡}}\log D(\;f_{\theta }(u_{‡}^{\left(m\right)}))$. In addition to the adversarial loss, the generator can be trained using fidelity-based losses, such as Perceptual Loss or Structural Similarity Index, to ensure that the generated images $f_{\theta }(u_{‡}^{\left(m\right)})$ closely match the reference images in both appearance and structure. However, the adversarial nature of the training process can lead to instability (due to the mini–max game structure inherent in Nash equilibrium) and limited mode coverage (even with the development of various mode collapse mitigation strategies), potentially erasing diagnostically important features.

Unlike GANs, which indirectly guide the generator to create reference-like images through adversarial feedback from the discriminator, DMs directly generate reference-like images by starting with noisy inputs and applying a carefully designed, iterative denoising process. DMs are often considered more stable than GANs, as the perceptual quality of the generated images is directly controlled by the score function $\nabla_{u}\log\;p_{\theta }(u)$ [84,85]. Unlike generative models that learn a lower-dimensional embedding space (e.g. variational autoencoders), DMs operate in the original high-dimensional space of the data, focusing on iteratively refining noisy samples using learned score functions. This process requires sequential updates to refine image quality, making the process of generating each image computationally intensive. Since the score functions are learned from a mixture of Gaussian kernels centred at the training data, a diverse and representative dataset is essential. Achieving this in clinical practice is challenging due to various constraints, including limitations in data collection (such as ethical and privacy concerns), patient variability (encompassing anatomical and pathological differences) and statistical heterogeneity influenced by factors such as variability in patient positioning and scanning protocols.

In medical imaging, a major challenge with generative models is their tendency to suppress anomalous features, often mistaking them for noise or perturbations in reference images. Due to their heavy reliance on memorizing the data’s probability distribution, these models are inclined to replicate common patterns from the training data [86]. With rare cases typically under-represented, generative models frequently produce images that reflect typical cases, failing to capture the subtle variations necessary for detecting rare pathologies. This limitation is a key reason why many promising results from generative models remain confined to academic research, in contrast to the clinical success and adoption of supervised learning models like U-Net.

### (b) Exploring alternative techniques for image reconstruction without traditional formulas

Conventional CT image reconstruction methods of finding explicit grid-based representations often fall short when addressing the ill-posed and nonlinear inverse problems in low-dose dental CBCT imaging, especially when projection data are compromised by the presence of metallic implants. As discussed in the previous sections, when projection data deviate significantly from the range space of the forward model, conventional reconstruction methods like FBP and FDK can result in a severely degraded image, leading to the loss of crucial diagnostic information. In such situations, even the most advanced image restoration algorithms, including DL-based methods and $L^{1}$-regularizations, may struggle to recover the missing details if they start with this compromised image. This challenge underscores our motivation to explore alternative image reconstruction techniques that avoid relying on traditional reconstruction formulas such as FDK (4.1).

The key to overcoming the limitations of conventional methods is to ensure that the reconstruction process accurately adheres to the accurate Lambert–Beer law (2.4), taking into account the polychromatic nature of photon energy. The main challenge lies in simplifying the energy-dependent attenuation variations in (2.5) to make it computationally feasible to extract the tomographic image $u_{E_{0}}$, corresponding to attenuation at a fixed energy level $E_{0}$. The first mathematical analysis of artefacts caused by such mismatches was presented by Park *et al.* [87], where the artefacts were characterized using Fourier integral operators and wavefront sets. Building on this rigorous foundation, Park *et al.* [88] developed a novel geometric corrector that simplifies energy-dependent mismatches into an energy-integrated formula based on the geometry of dense materials, such as metal, bone and teeth. This method has proven highly effective in industrial CT applications, where the homogeneity of the imaging objects and high-energy photons allow for easier and more accurate segmentation of dense materials.

However, in medical applications, the accurate segmentation of dense objects from CT images remains a significant challenge, and the complexity of determining energy-related parameters to manage interactions between metals, bone and teeth further limits the method’s practical effectiveness in clinical settings. Therefore, a key challenge now is to eliminate the need for both segmentation and energy-related parameter determination.

Recently, Park *et al.* [19] addressed this issue by employing INRs, effectively removing the need for accurate segmentation and parameter estimation. Additionally, this approach offers a promising alternative by circumventing the need for both reconstruction formulas and explicit grid-based representations. Here is how INRs help solve the problem.

*Implicit representation of the CT image*: The predicted CT image u implicitly represented by a trainable neural network ${\text{MLP}}_{\theta }$, where the input is the position x and the output is the greyscale value of the CT image at that specific position. Here, the network ${\text{MLP}}_{\theta }$ is a multi-layer perceptron network with parameters θ. This neural network naturally introduces regularization through its architecture and the optimization process, effectively embedding implicit relationships between pixels or voxels. To address the underdetermined nature of the problem, the network can incorporate prior knowledge of anatomical structures, such as the spatial interconnections between the head, jaws and teeth.

*Physics-based loss function*: The network is trained using a loss function grounded in the source data and the fundamental physical principles in (2.2):


(4.4) $$\mathrm{Loss}(\theta )=∭\left|P(\varphi ,s,t)-\int_{\ell_{\varphi ,s,t}}{\hat{u}}_{\theta }(x,z)dl_{x,z}-{\hat{\zeta }}_{u,\theta }(\varphi ,s,t)\right|dsdtd\varphi ,$$

where ${\hat{u}}_{\theta }$ represents the linear component providing the CT image from the MLP network, and the expression ${\hat{\zeta }}_{u,\theta }$ represents a nonlinear supplementary component (associated with beam-hardening) designed to handle the unknown part $\zeta_{u}$ in (1.1), which is also produced by the MLP network. This approach bypasses the need for conventional reconstruction algorithms like FBP and FDK, providing a more flexible framework for addressing forward model mismatches.

The major contribution lies in formulating the energy-dependent varying attenuation component, represented by ${\hat{\sigma }}_{\theta }$, which is nonlinearly related to the linear component ${\hat{u}}_{\theta }$ itself. This is because beam-hardening effects are material-dependent, and the predicted CT image from ${\hat{u}}_{\theta }$ reflects the varying tissue densities. Due to this interdependence, the parameters θ are primarily shared between ${\hat{u}}_{\theta }$ and ${\hat{\sigma }}_{\theta }$, requiring them to be learned simultaneously. Hence, the output of the MLP network ${\text{MLP}}_{\theta }$ should be divided into two components: the linear part ${\hat{u}}_{\theta }(x,z)={\text{MLP}}_{\theta }^{\text{ linear}}(x,z)$ and the nonlinear component ${\hat{\sigma }}_{\theta }(x,z)={\text{MLP}}_{\theta }^{\text{ nonlinear}}(x,z)$. The goal is to train the network to map


(4.5) $${\mathrm{MLP}}_{\theta }:(x,z)\mapsto \left({\mathrm{MLP}}_{\theta }^{\text{linear }}(x,z),{\mathrm{MLP}}_{\theta }^{\text{nonlinear }}(x,z)\right)$$

directly from the projection data P. The challenging nonlinear part ${\hat{\zeta }}_{u,\theta }$ in (1.1) can be approximated by the following mathematical formula that is based on a result by Park *et al.* [19,88]:


(4.6) $${\hat{\zeta }}_{u,\theta }(\varphi ,s,t)=\ln\left(\frac{\sinh\left(\int_{\ell_{\varphi ,s,t}}{\mathrm{MLP}}_{\theta }^{\text{nonlinear }}(x,z)dl_{x,z}\right)}{\int_{\ell_{\varphi ,s,t}}{\mathrm{MLP}}_{\theta }^{\text{nonlinear }}(x,z)dl_{x,z}}\right).$$

**Figure 4.**
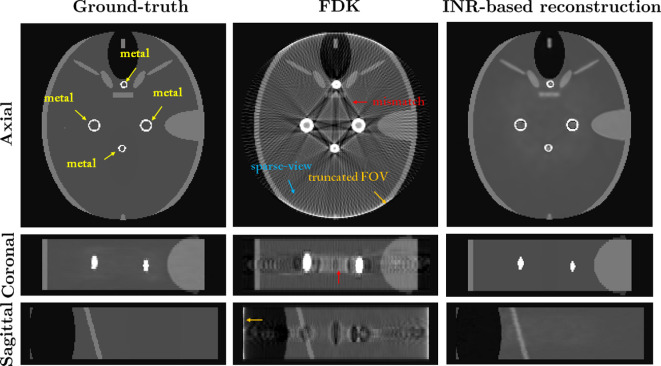
Performance of the INR-based method on simulated three-dimensional dental CBCT data. The three-dimensional images, with dimensions 200×200×30, are reconstructed from 200 projection views using a detector size of 300×90. The first column shows the ground truth three-dimensional image containing multiple metals. The second column shows the three-dimensional image reconstructed with the FDK algorithm, which suffers from severe streaking and shadowing artefacts caused by model mismatch, sparse-view sampling and a truncated FOV. The third column presents the results of the INR-based method, which significantly reduces these artefacts while preserving structural details around the metals.

Figure 4 visually compares the three-dimensional CBCT images reconstructed by the FDK and INR-based methods. In the second column of figure 4, the FDK-reconstructed images suffer from streaking and shadowing artefacts due to data insufficiency, resulting from FOV truncation, sparse views and model mismatch caused by the polychromatic nature of X-ray beams. By contrast, as shown in the third column of figure 4, the INR-based method significantly reduces these artefacts. The use of MLPs within the INR framework allows for the seamless integration of prior knowledge, enabling both local and global interactions among grid elements during the reconstruction process. Additionally, the INR-based method automatically extracts the energy-dependent attenuation component in an integrated manner, further enhancing image quality.

Despite its potential, further improvements are required for clinical application. The current model, as well as our simulation in figure 4, does not account for photon starvation, scattering caused by metal objects or motion artefacts from the slow rotation speeds of the dental CBCT systems. These factors can significantly compromise the accuracy and reliability of the reconstructed images. Motion artefacts will be discussed in more detail in the following section. While the INR method is designed for three-dimensional CBCT, applying it to a three-dimensional cone-beam transform is a time-consuming process, as it requires dividing each ray into N evenly spaced bins and uniformly sampling one point from each bin. In our simulation, it took over an hour to generate three-dimensional CBCT images with a resolution of 200×200×30. Moreover, under conditions of highly insufficient data, the INR method exhibits instability, with results fluctuating depending on the random initial weights, sometimes producing excellent reconstructions and other times yielding suboptimal outcomes.

Before closing this subsection, we briefly mention other INR-based approaches [20,21] that employ MLP networks to predict multiple energy-level attenuation coefficients $u_{E}=\left[u_{E_{1}},u_{E_{2}},\ldots ,u_{E_{N}}\right]$, which are subsequently used to generate a predicted sinogram incorporated into the loss function. In this framework, the MLP either directly predicts $u_{E}$ [20] or estimates a density from which $u_{E}$ are derived [21]. However, both approaches depend on prior knowledge of the incident X-ray energy spectrum η and the geometry or material composition of the metal, which can be challenging to obtain with high accuracy in clinical settings. Apart from MAR applications, INR techniques have also been used in sparse-view CT [18,89–93].

### (c) Challenges for motion artefacts

One of the primary challenges in dental CBCT is managing motion-induced artefacts [94–97]. Dental CBCT typically requires longer scan times, ranging from 7 to 24 s per rotation, compared with MDCT systems, which achieve rotation times of less than 0.3 s. This extended scan time is an unavoidable trade-off that makes dental CBCT systems significantly more cost-effective than conventional MDCT. However, despite the use of patient head fixation devices, the prolonged scan duration increases susceptibility to motion artefacts from movements such as breathing, swallowing or slight head shifts, potentially leading to data misregistration. As a result, effectively addressing motion artefacts remains a critical challenge in dental CBCT.

Traditional motion compensation methods that rely on motion models and parameter estimation are often ineffective in clinical settings due to their high computational demands, sensitivity to noise and limited ability to manage the wide range of motion artefacts caused by realistic patient movements. Given these complexities, image-based motion correction techniques using DL to capture motion features offer a more practical and efficient alternative, bypassing the challenges posed by intricate non-rigid motion models [96].

To evaluate the potential of DL-based motion correction for three-dimensional dental CBCT, we tested a neural network designed to predict the residual between motion-affected and motion-free two-dimensional projection images. This was achieved using two-dimensional reprojected images derived from motion-affected three-dimensional CBCT images at each scanning view. The network utilized the deep convolutional framelet architecture [98] for this purpose.

**Figure 5.**
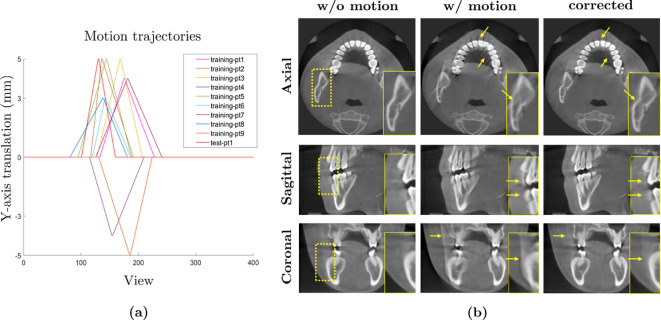
DL-based motion artefact correction for three-dimensional dental CBCT. (*a*) Ten plots depict simulated patient motion patterns as translations along the *y*-axis relative to scanning views, with nine representing training datasets (nine subjects) and one for the test dataset (one subject), showing displacements ranging from −5 to 5 mm randomly occurring between views 80 and 140. (*b*) The first column shows CT images reconstructed from motion-free data for the test subject, the second column shows images reconstructed from motion-affected data and the third column shows results from the DL-based correction method. As indicated by the yellow arrows, the DL method effectively reduced motion blurs near the bones and teeth.

Figure 5 illustrates the experimental results. This experiment employed 10 fully anonymized three-dimensional CBCT images of size 800×800×400, acquired using a commercial CBCT scanner (Q-FACE, HDXWILL, South Korea), with patient consent for research and development purposes. The images were resized to 200×200×100 for computational efficiency. As shown in figure 5*a*, we simulated a simple patient motion pattern as a linear translation along the *y*-axis, with displacements ranging from −5 to 5 mm occurring randomly between views 80 and 140 out of a total of 400 views. While individual projections appeared motion artefact-free during scanning, the entire set of projections became inconsistent in the CT sinogram due to patient motion. This inconsistency resulted in prominent motion artefacts in the reconstructed CT images using the FDK algorithm, as seen in the second column of figure 5*b*. The DL-based approach effectively corrected these distortions, as demonstrated in the third column of figure 5*b*, highlighting its potential to mitigate motion artefacts in dental CBCT imaging.

Despite these promising results, significant opportunities for improvement remain. Given the three-dimensional nature of dental CBCT and its high voxel dimensions, it is essential to evaluate the computational feasibility of DL methods, ensuring they can efficiently handle these high-dimensional datasets. For a practical motion correction solution, the method must be fast, nearly real-time and adaptable to various motion artefacts encountered in different clinical scenarios. It also needs to accommodate offset detectors and low-dose, localized CBCT systems commonly used in dental practices.

## 5. Discussion and conclusions

Dental CBCT, a highly cost-effective alternative at less than 10% of the price of standard multi-detector CT systems, has seen widespread adoption in oral and maxillofacial imaging within dental clinics over the past decade. However, while dental CBCT offers advantages such as lower cost, reduced radiation dose and a smaller physical footprint, these benefits inevitably come with several drawbacks, including increased noise (due to Compton scattering from the short distance between the body and detector), motion artefacts (caused by longer scan times), cone-beam artefacts (resulting from the violation of Tuy’s data completeness condition) and scanner-related artefacts such as truncated FOV and offset detectors [11]. These issues are further exacerbated by the presence of metallic implants, common in older patients, which introduce beam-hardening artefacts that compound the aforementioned challenges.

Given the cost constraints in advancing hardware technology (due to the need to maintain low cost, low dose and compact design), the focus has increasingly shifted towards developing advanced software techniques to enhance image quality. As discussed in §§2 and 4a, relying on the FDK (or FBP) algorithm as the basis, coupled with two-dimensional image-based priors such as generative models and sparsity-based regularization, has yielded only modest improvements. To overcome these barriers, it would be desirable to develop three-dimensional restoration techniques that fully leverage the inherent connectivity of anatomical structures along the *z*-axis, where cross-sectional images are highly correlated, with only small, gradual changes in anatomy from one slice to the next. Additionally, as discussed in §2c, it is advisable to move away from conventional backprojection-based reconstruction formulas, as severe data mismatches with the CT forward model can lead to irreparably degraded images, making them prohibitively difficult to correct even with advanced DL-based models used as post-processing tools.

In this context, the INR-based reconstruction methods discussed in §4b offer one of the promising alternatives by eliminating the reliance on conventional methods that require the use of the inverse operator of the forward model. Each two-dimensional X-ray projection captured from different angles provides relatively clear images, even in scenarios where tomographic images reconstructed through backprojection are severely degraded. This severe degradation primarily arises from inconsistencies between projection angles rather than from the quality of the individual two-dimensional X-ray projections. Additionally, the use of INR-based methods may eliminate cone-beam artefacts, which are commonly encountered in traditional approaches. While preliminary experiments suggest that INR methods may improve image quality, they are still in an early stage of development. Continued research is needed to enhance their robustness and generalizability, particularly in areas such as the design of loss functions that account for patient motion during scans. In addition, extensive experimental evaluation across diverse clinical scenarios is required to rigorously assess their performance for routine clinical use.

There is room for the development of generative models using unpaired data. As discussed in §4a, learning an accurate prior is challenging due to the diversity and stochastic heterogeneity in medical data. While supervised learning is ideal, obtaining paired data is nearly impossible. We suggest a hybrid unpaired–paired learning approach: initially, the model generates results using unpaired learning, and only the accurate outcomes are retained while unsatisfactory ones are discarded. These accurate results then serve as paired data for supervised learning. This iterative process is repeated, gradually accumulating paired data and progressively enhancing the model’s performance.

Finally, we would like to suggest considering a recently emerging alternative perspective in medical imaging systems: while traditional efforts have focused on cutting-edge advancements, there is increasing interest in developing lower-cost alternative systems, even if they produce lower-quality data. High-end CT and magnetic resonance imaging (MRI) machines involve high upfront costs, further compounded by maintenance, calibration and the need for specialized staff. These factors significantly contribute to rising healthcare costs and often lead to the overuse of expensive imaging technologies, exacerbating insurance and reimbursement challenges [1]. On the other hand, portable low-field MRI scanners and portable CT systems, although providing lower-quality data, offer significant advantages in terms of lower costs and improved accessibility to rural areas. These low-cost systems introduce the challenge of handling more ill-posed problems, requiring deeper mathematical analysis and innovative methodologies.

## Data Availability

All supporting data and code related to the performance of the INR algorithm, as presented in figure 3, are included as electronic supplementary materials with this submission to ensure transparency and reproducibility. Supplementary material is available online [99].
